# Antibiotic Resistance of *Escherichia coli* Serotypes from Cochin Estuary

**DOI:** 10.1155/2012/124879

**Published:** 2012-09-12

**Authors:** Divya P. Sukumaran, Srinivasan Durairaj, Mohamed Hatha Abdulla

**Affiliations:** ^1^Department of Marine Biology, Microbiology and Biochemistry, School of Marine Sciences, Cochin University of Science and Technology, Lakeside Campus, Cochin 682 016, India; ^2^Mathematics & Sciences Division, Richland Community College, One College Park, Decatur, IL 62521, USA; ^3^MacMurray College, 447 East College Avenue, Jacksonville, IL 62650, USA; ^4^Environment Genomics Laboratory, Civil and Environmental Engineering, Michigan State University, East Lansing, MI 48824, USA

## Abstract

This study aimed at detecting the prevalence of antibiotic-resistant serotypes of *Escherichia coli* in Cochin estuary, India. *E. coli* strains were isolated during the period January 2010–December 2011 from five different stations set at Cochin estuary. Water samples from five different stations in Cochin estuary were collected on a monthly basis for a period of two years. Isolates were serotyped, antibiogram-phenotyped for twelve antimicrobial agents, and genotyped by polymerase chain reaction for *uid* gene that codes for **β**-D-glucuronidase. These *E. coli* strains from Cochin estuary were tested against twelve antibiotics to determine the prevalence of multiple antibiotic resistance among them. The results revealed that more than 53.33% of the isolates were multiple antibiotic resistant. Thirteen isolates showed resistance to sulphonamides and two of them contained the *sul* 1 gene. Class 1 integrons were detected in two *E. coli* strains which were resistant to more than seven antibiotics. In the present study, O serotyping, antibiotic sensitivity, and polymerase chain reaction were employed with the purpose of establishing the present distribution of multiple antibiotic-resistant serotypes, associated with *E. coli* isolated from different parts of Cochin estuary.

## 1. Introduction

The emergence of *Escherichia coli* isolates with multiple antibiotic-resistant phenotypes, involving coresistance to four or more unrelated families of antibiotics, has been previously reported and is considered a serious health concern [1–3]. Antimicrobials are often used for therapy of infected humans and animals as well as for prophylaxis and growth promotion of food producing animals. Many findings suggest that inadequate selection and abuse of antimicrobials may lead to resistance in various bacteria and make the treatment of bacterial infections more difficult [4]. Antimicrobial agents can be found in sewage effluents, particularly in places where these drugs are extensively used, such as hospitals, pharmaceutical production plants, and around farms where animal feed contains these agents. It has been suggested that resistance in bacterial populations may spread from one ecosystem to another [5]. The wild dissemination of antimicrobial resistance among bacterial populations is an increasing problem worldwide. 

Antimicrobial resistance in *E. coli* has been reported worldwide. Treatment for *E. coli* infection has been increasingly complicated by the emergence of resistance to most first-line antimicrobial agents, including fluoroquinolones [6]. Sulfamethoxazole in combination with trimethoprim (cotrimoxazole) is still commonly used in human medicine for the treatment of urinary tract infections [7]. Resistance to at least two classes of antimicrobial agents in *E. coli *is nowadays an ordinary finding in human and veterinary medicine and has an increasing impact on available therapeutic options.

Serotyping of the antigens is a very useful method for detecting pathogenic *E. coli* strains in clinical specimens, foods, and environmental samples and for understanding the epidemiology of the pathogen [8]. According to the modified Kauffman scheme, *E. coli* is serotyped on the basis of its O (somatic), H (flagellar), and K (capsular) surface antigen profiles [9]. A total of 170 different O antigens, each defining a serogroup, are recognized currently. The O antigen, as part of the LPS (Lipopolysaccharide) in the outer membrane of Gram-negative bacteria, is a major target of both the immune system and bacteriophages and plays an important role in the bacterium-host interplay. It is one of the most variable cell constituents and also plays an important role in virulence [10].

Cochin estuary, a part of Vembanad Lake, an important Ramsar site supports good shellfish and finfish fishery. Cochin estuary, a typical tropical estuary, had undergone considerable pollution in the last decade resulting mainly from the development of satellite townships all across the estuary. The development is mostly in the housing sector as there is great demand for water front apartments. Most of the markets situating along the coasts of the estuary release their waste water directly into natural water body. *E. coli *being a typical faecal indicator organism offers an excellent means to look at the organic pollution that is taking place in the Cochin estuary. Since the estuary supports good shellfish and finfish resources, it forms the lifeline of large number of people directly and indirectly depending on it for fishing, recreation, and transportation. The increasing prevalence of pathogenic *E. coli* with multiple antibiotic resistance could have a bearing on fish and shellfish harvested from the estuary which indirectly affect the health of people. The present study has been carried out with an objective of estimating the diversity of *Escherichia coli* serotypes from Cochin estuary and the risk posed by them is evaluated in terms of antibiotic resistance.

## 2. Materials and Methods

### 2.1. Description of the Study Area

**Figure 1:**
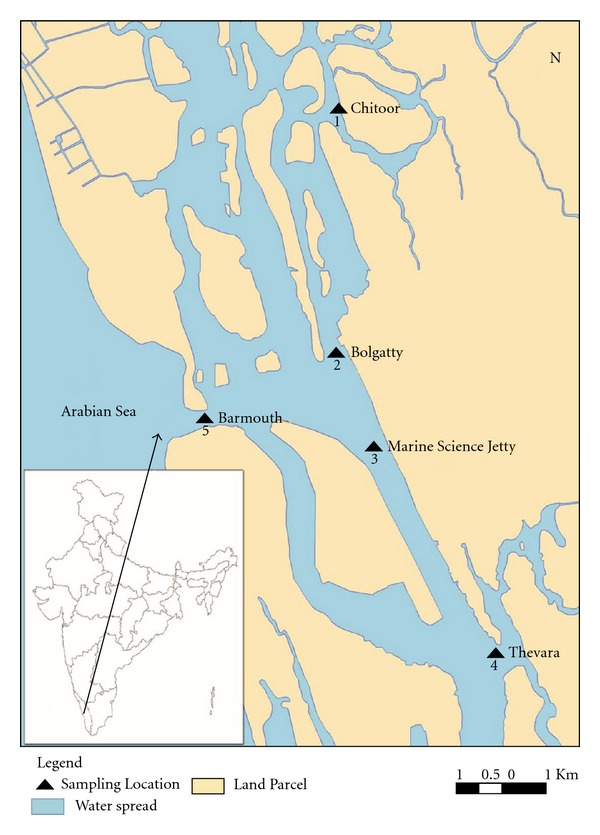


The water samples were collected from five different stations along Cochin estuary (Figure 1). The stations were selected based on their closeness to satellite townships and waste inputs. Two of the stations such as Chittoor (station 1) and Thevara (station 4) were fixed upstream, two in the central part of the estuary, namely, Bolgatty (station 2) and Marine Science Jetty (station 3), and one at the Barmouth (station 5). The sampling stations were fixed in and around Cochin estuary as they were suspected to receive high levels of sewage inputs.

### 2.2. Collection of Samples

The water samples were collected monthly from five different stations along Cochin estuary for a period of two years from January 2010 to December 2011. The water samples were collected in sterile plastic bottles (Tarson, India) one foot below the surface to get a better representation of the water column. Water samples were transported to the laboratory in an ice box and subjected to bacteriological examination within four hours of collection.

### 2.3. Isolation, Identification, and Serotyping of **E. coli**

Samples were analysed for faecal coliforms by most probable number method. The most probable number (MPN) load of faecal coliform bacteria was determined by three-tube dilution method using MacConkey broth as medium. Ten mL, 1 mL, and 0.1 mL of water samples were inoculated into respective dilution tubes containing inverted Durham's tubes. Inoculated tubes were incubated at 37°C for 24 hours and observed for growth and gas production. For isolation of *E. coli*, one loopfuls from positive MacConkey broth tube cultures were streaked onto Eosin methylene blue (EMB) (Hi-Media, India) plates and incubated at 37°C for 24 Hours. After incubation, plates were examined, typical *E. coli-*like colonies were selected and subcultured. All isolates were submitted to a biochemical screening which included the Indole test, Methyl Red test, Voges-Proskauer test, and Citrate utilization (IMViC) test. The cultures giving + + − − reaction were confirmed as *E. coli*. Confirmed *E. coli* cultures were serotyped at National *Salmonella* and *Escherichia* Center, Central Research Institute, Kasauli, Himachal Pradesh, India.

### 2.4. Isolation of DNA from **E. coli**

DNA from the bacterial genome was extracted as per standard Proteinase-K digestion method [11]. Bacterial cultures were suspended in Luria Bertani broth (Hi-media, India) and incubated in an orbital incubator (Orbitek, India) at 37°C, 110 rpm for 12-hours. The 12-hour old bacterial cells were pelleted at 15000 g for 10 minutes and then suspended in TEN (Tris-HCl (pH 7.2), 10 mM EDTA, 250 mM NaCl) buffer having 1% sodium dodecyl sulphate (Hi-Media, India). Proteinase-K (GeNei, India) was then added to a final concentration of 100 *μ*g/mL and mixed gently. The suspension was incubated at 37°C for 60 min. DNA obtained by sequential phenol-chloroform and chloroform-isoamyl alcohol extractions was precipitated by adding 2.5 volumes of absolute ethanol, and DNA was suspended in 100 *μ*L of TE buffer (10 mM Tris-HCl, 1 mM EDTA-pH 7.5). DNA was checked for purity by Agarose gel electrophoresis.

### 2.5. Polymerase Chain Reaction (PCR) for Detection of *uid A* Gene in **E. coli**

The polymerase chain reaction (PCR) was used to detect the presence of the *uid* gene, which codes for the *β*-D-glucuronidase enzyme. A 147 bp coding region of the* E. coli uid *gene was amplified by PCR, using the 20 and 21-mer primers UAL-754 (5′-AAAACGGCAAGA AAAAGCAG-3′) and UAR-900 (5′-ACGCGTGGTTACAGTCTTGCG-3′) [12]. The optimized protocol was carried out with a PCR mix of 25 *μ*L that contained 2.5 mM MgCl_2_, 2.5 *μ*L of Taq buffer (Tris (pH 9.0) at 25°C, KCl, and Triton X-100), 2.5 mM each of dNTP mixture, 1 pmol/*μ*L of each of the primers, 1 U of Taq polymerase (GeNei, India), and 1 *μ*L of the DNA template. Amplification was performed with a thermal cycler programmed for 1 cycle of 2 min at 94°C; 25 cycles of 1 min at 94°C, 1.5 min at 58°C, 2 min at 72°C; 1 cycle of 5 min at 72°C. PCR products were then electrophoresed on a 1.5% agarose gel (Hi-Media, India), stained with ethidium bromide (GeNei, India), and visualized by Gel Documentation System (Bio-Rad Gel Doc EZ Imager, USA).

### 2.6. Antimicrobial Susceptibility Testing

Antibiograms and their interpretation were made using the disk diffusion method [13], following the Clinical and Laboratory Standards Institute (CLSI) guidelines [14]. All *E. coli* isolates were examined for resistance to ampicillin (Amp, 10 mcg), amikacin (Ak, 30 mcg), ceftriaxone (Ctr, 30 mcg), chloramphenicol (C, 30 mcg), ciprofloxacin (Cip, 5 mcg), cotrimoxazole (Cot, 25 mcg), gentamicin (Gen, 10 mcg), kanamycin (K, 30 mcg), nalidixic acid (Na, 30 mcg), streptomycin (S, 10 mcg), tetracycline (Te, 30 mcg), and trimethoprim (Tr, 5 mcg). 

### 2.7. PCR Detection of *sul 1 *Gene

 The presence of *sul 1* gene was detected using the PCR method described by Marynard et al. [3]. The primers used for *sul 1* gene were 5′-TTCGGCATTCTGAATCTCAC-3′ and 5′-ATGATCTAACCCTCGGTCTC-3′. Amplified PCR products were separated using 1.5% agarose gel (Hi-Media, India), stained with ethidium bromide (GeNei, India), and visualized by Gel Documentation System (Bio-Rad Gel Doc EZ Imager, USA).

### 2.8. Detection of Class 1 Integrons

 The presence of class 1 integrons was detected using the PCR method described by Levesque et al. [15]. Class 1 integrons were amplified using degenerate primers 5′ CS (5′-GGC ATC CAA GCA GCA AG-3′) and 3′ CS (5′-AAG CAG ACT TGA CCT GA-3′). PCR products were then electrophoresed on a 1.5% agarose gel (Hi-Media, India), stained with ethidium bromide (GeNei, India), and visualized by Gel Documentation system (Bio-Rad Gel Doc EZ Imager, USA).

## 3. Results

Serotyping of the 75 *E. coli *isolates that were from Cochin estuary revealed that they belonged to 25 different serotypes (Table 1). Twelve strains of *E. coli *were rough and hence not typable. All isolates were confirmed by molecular level identification by polymerase chain reaction with primers UAL-754 and UAR-900 to amplify the amino coding region of *uid A *produced amplified DNA bands of 147 bp for all *E. coli* isolates. Stations close to the city such as Bolghatty and Off Marine Science Jetty yielded more diverse serotypes of *E. coli*. 

The results (Table 1) revealed remarkable diversity of *E. coli* strains in the system. The diversity of *E. coli* serotypes was higher at station 2 (32%) followed by station 3 (22%), station 5 (17%), station 4 (15%), and station 1 (13%). *E. coli* serotypes such as O4, O5, O60 were isolated with high frequency, whereas O157, O153, O148 were encountered very rarely. The serotypes commonly associated with pathogenic strains, such as O5, O4, and O41, were found significantly high at station 2 (Bolgatty) (Table 2). 

**Figure 2:**
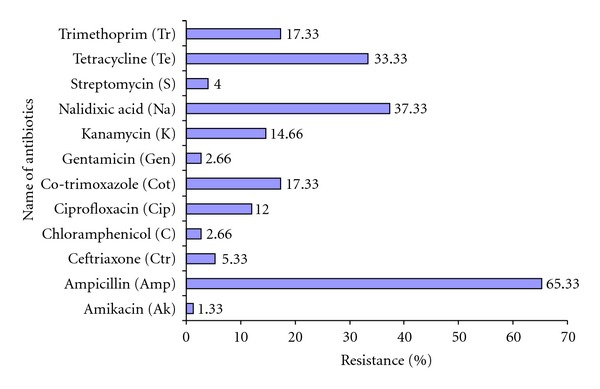
Percentage of antibiotic resistance among *Escherichia coli* strains from Cochin estuary during the study period (January 2010–December 2011).

Percentage of antibiotic resistance of *E. coli* strains isolated from Cochin estuary is given in Figure 2. Most of the *E. coli *strains were found to be resistant to ampicillin (65.33%) followed by nalidixic acid (37.33%), tetracycline (33.33%), cotrimoxazole (17%), trimethoprim (17%), kanamycin (14%), and ciprofloxacin (12%). The least resistance was detected against ceftriaxone (5.33%), streptomycin (4%), chloramphenicol (2.66%), gentamicin (2.66%), and amikacin (1.33%). The *E. coli* strains showing resistance to five or more antimicrobial agents belongs to serotypes O5, O4, O60, O69, O1, O157, O20, O153, O22, and O34. Nearly 60% and 50% of *E. coli* isolates encountered at stations 3 and 2, respectively, were resistant to more than five antibiotics. While 18% of *E. coli* isolates recovered from station 4 were resistant to more than five antibiotics, none of the isolates from station 5 was resistant to more than five antibiotics. One *E. coli *isolate recovered from Chittor (station 1) was resistant against more than five antibiotics.

A total of 24* E. coli *strains were isolated from station 2 (Bolgatty). All isolates were susceptible to amikacin, chloramphenicol, gentamicin, and streptomycin. About 29% of *E. coli* strains isolated from Bolgatty showed resistance against the sulphonamides tested. The *E. coli* strains isolated from Off Marine Science Jetty were resistant to ampicillin (76%); some were resistant to tetracycline (41%), nalidixic acid (41%), kanamycin (35%), and ciprofloxacin (17%). One *E. coli* isolate was resistant to cotrimoxazole and trimethoprim, and another isolate was resistant to chloramphenicol. A total of 11 strains were recovered from station 4 (Thevara). All *E. coli* were susceptible to amikacin, chloramphenicol, ciprofloxacin, gentamicin, and streptomycin. Almost 81% of the isolates showed ampicillin resistance and 27% isolates exhibited tetracycline resistance. While all isolates were susceptible to amikacin, ceftriaxone, chloramphenicol, ciprofloxacin, cotrimoxazole, gentamicin, kanamycin, streptomycin, and trimethoprim, resistance to ampicillin, nalidixic acid, and tetracycline ranged from 15% to 30%.

**Figure 3:**
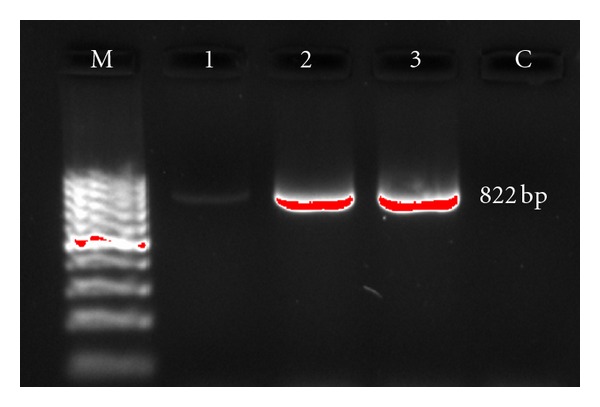
PCR detection of the *sul 1* gene in* E. coli *isolates. Lanes: M, 100 bp ladder; 1, clinical strain; 2, ES11; 3, ES44; C, negative control.

**Figure 4:**
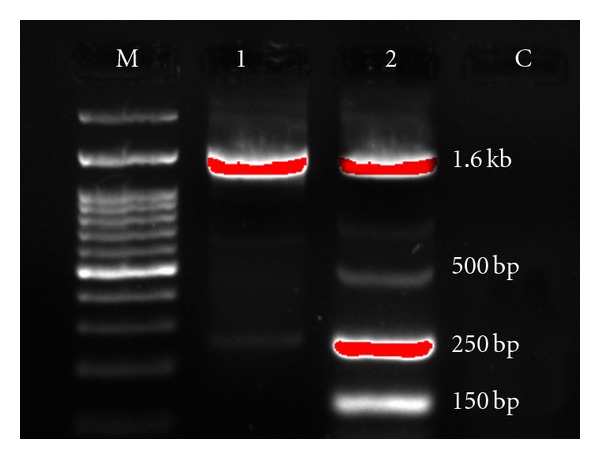
PCR detection of the class 1 integron in* E. coli *isolates. Lanes: M, 100 bp ladder; 1, ES44; 2, ES11; C, negative control.

Of 75 strains of *E. coli*, 13 were resistant to sulphonamides. Of these, 2 were positive in PCR for *sul 1* gene (Figure 3). Both isolates were highly resistant to sulphonamides; one strain (ES11) was also resistant to ampicillin, ciprofloxacin, ceftriaxone, cotrimoxazole, gentamicin, kanamycin, nalidixic acid, tetracycline, and trimethoprim; the other (ES44) was also resistant to ampicillin, ciprofloxacin, cotrimoxazole, nalidixic acid, streptomycin, tetracycline, and trimethoprim.  Figure 4 shows the PCR amplification of the class 1 integrons from environmental isolates. Amplicons with the sizes of 1.6 Kb, 500 bp, 250 bp, 150 bp were obtained in ES11 while ES44 gave the product of 1.6 Kb size. These results showed that the strains tested contain an integron which possessed one or more inserted genes, suggesting the presence of multiresistance integrons in these strains.

## 4. Discussion

*E. coli* causes a wide variety of intestinal diseases. Diarrheagenic *E. coli* is classified into six categories: enterotoxigenic *E. coli* (ETEC), enteropathogenic *E. coli *(EPEC), enterohemorrhagic *E. coli *(EHEC), enteroaggregative *E. coli *(EAEC), enteroinvasive *E. coli* (EIEC), and diffusely adherent *E. coli* (DAEC) [9]. Bej et al. [12] suggested that a PCR-based method for *uid A* gene is more sensitive in detecting *E. coli* isolates from water samples. The *uid* gene was detected in all *E. coli* isolates. According to the result of serotyping, O4 (12%) was predominant, followed by O5 (8%), O60 (8%), R (6.6%), O41 (5.3%), O59, O1 (4%), O22, O21, O102, O103, O116, O69, O91 (2.6%), and O157, O34, O35, O37, O20, O141, O103, O104, O49, O148, O64, O153 (1.3%). When an analysis of the geographic distribution of *E. coli* O157 was done by Sehgal et al. [16], it was observed to be widely distributed in all parts of India showing wide prevalence of this strain in almost all regions of the country and 1.1% of O157 isolates were from Kerala. Though food- and water-borne diarrheal diseases are very common in the study area, the investigations are usually limited to characterisation of *E. coli* alone. Serotyping of the strains is not undertaken by most clinics. Hence it is difficult to comment on the prevalence of these strains from the clinical samples in Cochin region. 

Perelle et al. [17] reported that contamination by the pathogenic *E. coli* serotypes, including O103, O157, and O145, represents a major public health concern. The isolation of a great number of strains with the serotypes O5, O1, and O104 was a reason of concern because they have been recognised as pathogenic *E. coli* serotypes. Conventional serotyping methods for* E. coli *somatic and flagellar antigens are still important technique in many laboratories for diagnosis and surveillance [18]. Furthermore, serologic antigens are not directly involved in virulence but can provide important information about the circulating serotypes in the communities and in outbreaks [9]. 

The interesting observation was that more diverse serotypes were isolated from the station near to Cochin city, which suggests the possible release of these organisms through hospital waste from many of the hospitals in and around Cochin City. Hospital wastewater is often contaminated by antimicrobial agents, which even in subinhibitory concentrations may promote selection and survival of resistant strains [19, 20]. These serotypes may represent a significant risk to human to acquire severe infections and might emerge as a major public health problem in our country. 

A total of 75* E. coli* strains isolated from five different stations in Cochin estuary were tested against 12 different antibiotics. Though there was spatial variation in the antibiotic resistance pattern, all the *E. coli* strains were resistant to two or more antimicrobials in different combinations. In general, the resistance to sulphonamides is increasing in incidence among *E. coli* serotypes from the study area. Previous studies from our research group [21] also showed high prevalence of antibiotic resistance in *E. coli* isolates from same region, although resistance levels were higher than the ones obtained in the present study. Antimicrobial drug therapy is recommended when diarrhoeal disease is severe to reduce duration of the symptoms. Concerns are increasing in studies reporting high levels of resistance to ampicillin, chloramphenicol, and trimethoprim/sulfamethoxazole among diarrhoea-associated bacteria [22]. *Enterobacteriaceae *showing resistance to nalidixic acid and susceptibility to ciprofloxacin presents a mutation in the A subunit of the DNA-Gyrase, usually, at a position equivalent to amino acid 83 in *E. coli *[23–25]. 

Different use patterns of antimicrobial agents are expected to have some impact on the distribution of antimicrobial resistance phenotypes [26] and possibly of resistant determinants. The result of the antibiotic resistance analysis revealed that more than 53.33% of *E. coli* were multiple antibiotic resistant. Detection of an isolate (ES11) simultaneously resistant to 10 antimicrobials is a concern, as it may pose risk to public health where these isolate to gain to the food chain. Serotypes showing multidrug-resistance to five or more antibiotics have been reported [27]. Integron-positive strains showed resistance against different groups antimicrobial agents (cephalosporins, aminoglycosides, quinolones, sulphonamides, tetracycline) tested. Isolates ES11 and ES44 were screened for class 1 integrons. Both of the isolates carried class 1 integrons and sul I gene, which is consistent with the presence of sul-associated class 1 integrons [28]. In *Escherichia coli*, trimethoprim-sulfamethoxazole resistance often correlates with the presence of dihydrofolate reductase (DHFR) and dihydropteroate synthase (DHPS) genes in integrons [29, 30]. Multiple antibiotic resistance may be acquired through mobile genetic elements such as plasmids, transposons, and class 1 integrons [27, 31]. Integrons play an essential role in facilitating the transfer of the resistance genes, contributing to the creation of multidrug-resistant phenotype [32]. 

We conclude that the high prevalence of different serotypes and multidrug resistance detected in our study is a matter of concern, since there is a large reservoir of antibiotic resistance genes within the community, and that the resistance genes and plasmid-encoded virulent genes were easily transferable to other strains. Pathogen cycling through food is very common and fish and shellfish that harbour these diarrheagenic strains might pose potential health risk to consumer. Cochin estuary supports good shellfish and finfish fishery which is exploited by local fishermen for livelihood. Shellfish being a filter feeder tend to concentrate bacteria in their body and lack of depuration practices in the study area adds guaranty to the problem of shellfish-borne food poisoning. The present study contributes to understanding the prevalence of different serotypes of *E. coli* and their antibiotic resistance patterns. In conclusion, we would like to highlight that the diversity of *E. coli* serotypes in the Cochin estuary has increased considerably when compared to our previous studies [21] which might be due to large-scale influx of organic wastes into the estuary from the satellite townships along the banks of Cochin estuary. It may also be worthwhile for the clinical laboratories of this locality to further characterise the *E. coli* serotypes isolated from diarrheal patients so as to get a clear picture of the emergence of pathogenic strains of *E. coli* in the study area. Future experimental studies to assess the efficiency of antimicrobial resistance transfer using different donor and recipient *E. coli* combinations would be of value to improve our understanding of how resistance genes may flow across strains.

## Figures and Tables

**Table 1:** Prevalence of different serotypes of *E. coli* in Cochin estuary during the study period January 2010–December 2011.

*E. coli* serotypes	Percentag of incidence	*E. coli* serotypes	Percentage of incidence
O4	12	O5	8
O60	8	R (Rough)	6.6
O41	5.3	O59	4
O1	4	O22	2.6
O21	2.6	O102	2.6
O103	2.6	O116	2.6
O69	2.6	O91	2.6
O157	1.3	O34	1.3
O35	1.3	O37	1.3
O20	1.3	O141	1.3
O104	1.3	O49	1.3
O148	1.3	O64	1.3
O153	1.3	UT (untypable)	16

**Table 2:** Spatial distribution of various serotypes of *E. coli* in Cochin estuary during the study period (January 2010–December 2011).

Station/name	*E. coli* serotypes encountered
(1) Chittor	O60 (1), O22 (1), O59 (1), O1 (2), O91 (2), O21 (1), R (2)
(2) Bolgatty*	O59 (1), O157 (1), O41 (2), O4 (7), O5 (4), O60 (1), O69 (2), O21 (1), O37 (1), O34 (1), R (1), UT (2)
(3) Off Marine Science Jetty*	O35 (1), O22 (1), O20 (1), O141 (1), O116 (1), O41 (1), O148 (1), O153 (1), O60 (1), R (1), UT (7)
(4) Thevara	O59 (1), O49 (1), O41 (2), O4 (1), O116 (1), O60 (3), R (1), UT (1)
(5) Barmouth	O1 (1), O4 (1), O5 (2), O64 (1), O102 (2), O103 (2), O104 (1), UT (2)

*Stations close to the Cochin city.

**Values in parenthesis indicate number of isolates of each serotype.

## References

[1] Ariza RR, Cohen SP, Bachhawat N, Levy SB, Demple B (1994). Repressor mutations in the *marRAB* operon that activate oxidative stress genes and multiple antibiotic resistance in *Escherichia coli*. *Journal of Bacteriology*.

[2] Cohen SP, McMurry LM, Hooper DC, Wolfson JS, Levy SB (1989). Cross-resistance to fluoroquinolones in multiple-antibiotic-resistant (*Mar*) *Escherichia coli* selected by tetracycline or chloramphenicol: decreased drug accumulation associated with membrane changes in addition to OmpF reduction. *Antimicrobial Agents and Chemotherapy*.

[3] Maynard C, Fairbrother JM, Bekal S (2003). Antimicrobial resistance genes in enterotoxigenic *Escherichia coli* O149:K91 isolates obtained over a 23-year period from pigs. *Antimicrobial Agents and Chemotherapy*.

[4] Kolář M, Urbánek K, Látal T (2001). Antibiotic selective pressure and development of bacterial resistance. *International Journal of Antimicrobial Agents*.

[5] Johnson JR, Sannes MR, Croy C (2007). Antimicrobial drug-resistant *Escherichia coli* from humans and poultry products, Minnesota and Wisconsin, 2002–2004. *Emerging Infectious Diseases*.

[6] Sabaté M, Prats G, Moreno E, Ballesté E, Blanch AR, Andreu A (2008). Virulence and antimicrobial resistance profiles among *Escherichia coli* strains isolated from human and animal wastewater. *Research in Microbiology*.

[7] Perreten V, Boerlin P (2003). A new sulfonamide resistance gene (*sul*3) in *Escherichia coli* is widespread in the pig population of Switzerland. *Antimicrobial Agents and Chemotherapy*.

[8] Wang Q, Ruan X, Wei D (2010). Development of a serogroup-specific multiplex PCR assay to detect a set of *Escherichia coli* serogroups based on the identification of their O-antigen gene clusters. *Molecular and Cellular Probes*.

[9] Nataro JP, Kaper JB (1998). Diarrheagenic *Escherichia coli*. *Clinical Microbiology Reviews*.

[10] Feng L, Perepelov AV, Zhao G (2007). Structural and genetic evidence that the *Escherichia coli* O148 O antigen is the precursor of the *Shigella dysenteriae* 1 O antigen and identification of a glucosyltransferase gene. *Microbiology*.

[11] Sambook J, Fritsch E, Maniatis T (1989). *Molecular Cloning: A Lboratory Manual*.

[12] Bej AK, DiCesare JL, Haff L, Atlas RM (1991). Detection of *Escherichia coli* and *Shigella* spp. in water by using the polymerase chain reaction and gene probes for *uid*. *Applied and Environmental Microbiology*.

[13] Bauer AW, Kirby WM, Sherris JC, Turck M (1966). Antibiotic susceptibility testing by a standardized single disk method. *American Journal of Clinical Pathology*.

[14] CLSI (2007). *Performance Standards for Antimicrobial Susceptibility Testing, 17th Informational Supplement. M100-S17*.

[15] Levesque C, Piche L, Larose C, Roy PH (1995). PCR mapping of integrons reveals several novel combinations of resistance genes. *Antimicrobial Agents and Chemotherapy*.

[16] Sehgal R, Kumar Y, Kumar S (2008). Prevalence and geographical distribution of *Escherichia coli* O157 in India: a 10-year survey. *Transactions of the Royal Society of Tropical Medicine and Hygiene*.

[17] Perelle S, Dilasser F, Grout J, Fach P (2007). Screening food raw materials for the presence of the world’s most frequent clinical cases of Shiga toxin-encoding *Escherichia coli* O26, O103, O111, O145 and O157. *International Journal of Food Microbiology*.

[18] Pérez C, Gómez-Duarte OG, Arias ML (2010). Diarrheagenic *Escherichia coli* in children from Costa Rica. *American Journal of Tropical Medicine and Hygiene*.

[19] Kim S, Jensen JN, Aga DS, Weber AS (2007). Tetracycline as a selector for resistant bacteria in activated sludge. *Chemosphere*.

[20] Al-Ahmad A, Daschner FD, Kümmerer K (1999). Biodegradability of cefotiam, ciprofloxacin, meropenem, penicillin G, and sulfamethoxazole and inhibition of waste water bacteria. *Archives of Environmental Contamination and Toxicology*.

[21] Chandran A, Hatha AAM, Varghese S, Sheeja KM (2008). Prevalence of multiple drug resistant *Escherichia coli* serotypes in a tropical estuary, India. *Microbes and Environments*.

[22] Nguyen TV, Le PV, Le CH, Weintraub A (2005). Antibiotic resistance in diarrheagenic *Escherichia coli* and Shigella strains isolated from children in Hanoi, Vietnam. *Antimicrobial Agents and Chemotherapy*.

[23] Ruiz J, Marco F, Goni P (1995). High frequency of mutations at codon 83 of the* gyr*A gene of quinolone-resistant clinical isolates of *Escherichia coli*. *Journal of Antimicrobial Chemotherapy*.

[24] Del Mar Tavío M, Vila J, Ruiz J, Ruiz J, Martín-Sánchez AM, Jiménez De Anta MT (1999). Mechanisms involved in the development of resistance to fluoroquinolones in *Escherichia coli* isolates. *Journal of Antimicrobial Chemotherapy*.

[25] Vila J, Ruiz J, Marco F (1994). Association between double mutation in *gyr*A gene of ciprofloxacin- resistant clinical isolates of *Escherichia coli* and MICs. *Antimicrobial Agents and Chemotherapy*.

[26] Aarestrup FM (1999). Association between the consumption of antimicrobial agents in animal husbandry and the occurrence of resistant bacteria among food animals. *International Journal of Antimicrobial Agents*.

[27] Mora A, Blanco JE, Blanco M (2005). Antimicrobial resistance of Shiga toxin (verotoxin)-producing *Escherichia coli* O157:H7 and non-O157 strains isolated from humans, cattle, sheep and food in Spain. *Research in Microbiology*.

[28] Recchia GD, Hall RM (1995). Gene cassettes: a new class of mobile element. *Microbiology*.

[29] Huovinen P, Sundstrom L, Swedberg G, Skold O (1995). Trimethoprim and sulfonamide resistance. *Antimicrobial Agents and Chemotherapy*.

[30] White PA, McIver CJ, Rawlinson WD (2001). Integrons and gene cassettes in the *Enterobacteriaceae*. *Antimicrobial Agents and Chemotherapy*.

[31] Singh R, Schroeder CM, Meng J (2005). Identification of antimicrobial resistance and class 1 integrons in Shiga toxin-producing *Escherichia coli* recovered from humans and food animals. *Journal of Antimicrobial Chemotherapy*.

[32] Shaheen BW, Oyarzabal OA, Boothe DM (2010). The role of class 1 and 2 integrons in mediating antimicrobial resistance among canine and feline clinical *E. coli* isolates from the US. *Veterinary Microbiology*.

